# Maternity and Mothering

**Published:** 1911-10-21

**Authors:** 


### MATERNITY AND MOTHERING.

(Inquiries and Correspondence are invited)

### The Care of Children's Teeth.

There are few, if any, respects in which the improved hygiene of the upper and middle classes during the last twenty years is more apparent than in connection with the care of the teeth. It is realised now by the majority of educated parents that their offspring must be made to carry out measures intended to preserve both the temporary and permanent sets, and must be taken at regular intervals to a competent dentist for advice and treatment. There are, of course, children in whom entire neglect of the teeth is not followed by any special deterioration of their dentures, just as in past times there have always been some people with perfect sets. But in general, for nineteen people out of twenty the condition of the jaws and teeth in adult life is a reliable index of the attention which has been bestowed on oral hygiene by their parents in early life.

Among the labouring classes, too, advances have been made in the dissemination of the truth by precept in schools, hospitals, missions, clubs, etc. But, broadly speaking, it is still true that the poor of this kingdom take little or no care of their mouths, and that dental caries and all its attendant evils are some of the principal causes of physical ill-health and inefficiency among the half-educated classes who constitute the great bulk of our population. It is no exaggeration to say that few missionary enterprises are more pregnant with health and happiness and more urgently required for the future well-being of the nation than that which aims at the total prevention of dental caries. That such a consummation is easily possible no one can doubt; that so little is being done to attain it everyone must regret. If preventable, why not prevented? is a question which at present can only be answered with the blush of shame. Posterity will surely indict us for our criminal failure, as a people, to realise our responsibilities and to rise to them.

Taking for granted, therefore, that the ravages of dental caries (decayed teeth) are a reproach to our national honour and a serious drain on our national health and vigour, let us turn to what is being done and what more can be done for repairing the mischief and for preventing its further evil extension. First and foremost, what have dentists and doctors to suggest? They are our expert advisers in this matter, and on them rests the responsibility of devising a plan of campaign. It is due to the dentists to say that they have done and are doing their share. They have investigated and firmly established the causes of decay, and have worked hard not only to discover how it may be prevented, but also to disseminate that knowledge. The next generation, we may hope, will profit by these altruistic researches, which can only have the effect of reducing steadily the emoluments of the dental profession.

The medical profession sees constantly the awful effects of dental caries, pyorrhœa, and other results of that apathy in respect of care of the teeth which is almost universal except among the educated classes, and even among the latter is more widespread than it ought to be. To the doctors, then, the magnitude of the evil is fully manifest, and the importance of prophylaxis is most brought home. Are they, then, seconding to their utmost ability the efforts of the dentists? Unfortunately strict candour compels one to answer this question in the negative. It is true that a good many doctors are impressing on mothers the importance of attention to the children's teeth, and that these include the most progressive and well-informed members of the profession. But even amongst these medical men there is much ignorance of the true principles of oral hygiene. Many a medical man who prides himself on keeping carefully up to date, and can give a full account of solid carbon dioxide snow or of Wertheim's operation, is woefully behind the times on matters connected with the care of the teeth. With the most conscientious intentions of doing his duty to his infant patients, he may mislead parents, or at least fail to tell them the whole truth about the care of their babies' mouths and teeth. Besides these, who merely fail to do their whole duty through ignorance of a subject which they have probably never been taught, there are unfortunately many others too careless or too stupid to recognise the oral origin of their patients' diseases, and to preach the doctrines of cleanliness of mouth and teeth when they do meet with the lack of it. So that in the main it must be admitted that the medical profession is not doing all it should, though at the same time it is gradually awaking to a sense of its responsibilities.

To educate the public it is first of all necessary to educate the dentists and the doctors. The former have fairly well educated themselves, the latter still require some degree of missionary enterprise. It is, therefore, with pleasure that all those who take broad views of national health and administrative medicine will greet the appearance of a book by Mr. Sim Wallace,* the avowed object of which is to place before the medical profession in brief and well-digested form those results of dental research which are firmly enough established to serve as foundations for future advances and for present-day teaching. Since the conclusions thus presented are of the utmost value and importance to mothers and all who have the care of little children, no apology is needed for reproducing them in some detail; and in a future article it is our intention to explain Mr. Wallace's theories and how they bear on the everyday life of the child. Meanwhile we would emphasise once again the fact that decayed teeth are such a prolific source of ill-health that it is doubtful if even alcoholism exceeds dental caries in importance from a public health point of view.

* *The Prevention of Dental Caries*, by J. Sim Wallace, M.D., L.D.S. The Dental Record Office. 1911.

